# The complete chloroplast genome of *Epimedium muhuangense* (Berberidaceae)

**DOI:** 10.1080/23802359.2022.2071650

**Published:** 2022-05-16

**Authors:** Jing Wang, Ruoqi Huang, Qiong Liang, Yanjun Zhang

**Affiliations:** a Department of Botany, College of Forestry, Jiangxi Agricultural University, Nanchang, People’s Republic of China; b Key Laboratory of Plant Germplasm Enhancement and Specialty Agriculture, Wuhan Botanical Garden, Chinese Academy of Sciences, Wuhan, People’s Republic of China; c Department of Bioengineering, School of Life Sciences, University of Chinese Academy of Sciences, Beijing, People’s Republic of China

**Keywords:** *Epimedium muhuangense*, chloroplast genome, Berberidaceae

## Abstract

*Epimedium muhuangense* S. Z. He & Y. Y. Wang 2017, one of the rare unifoliolate species in the *Epimedium* genus of Berberidaceae, is distributed in the Guizhou province of China. In present research, we sequenced the complete chloroplast genome of *E. muhuangense* with Illumina sequencing technology. The whole genome was 157,264 bp in length, which consisted of a large single-copy region (LSC, 88,588 bp), a small single-copy region (SSC, 17,036 bp), and a pair of inverted repeat regions (IRa and IRb, 25,820 bp). A total of 112 unique genes were successfully annotated, consisting of 78 protein-encoding genes, 30 rRNA, and four tRNA. Phylogenetic analysis demonstrated that *E. muhuangense* is closely related to *E. elachyphyllum*.

*Epimedium* L. is the largest herbaceous genus of Berberidaceae and contains about 62 species. China is the distribution and diversity center of *Epimedium*, in which there are about 52 species with continued evolution (Luo et al. 2021; Guo et al. 2022). As the traditional Chinese medicines, *Epimedium* plants have been identified with many pharmacological activities, such as improving cardiovascular function, anti-cancer, anti-osteoporosis and anti-aging activities (Zhou et al. 2021).

However, *Epimedium* is taxonomically and phylogenetically regarded as one of the most challengingly difficult taxa in plants, since abundant morphological variations complicate the interspecific relationship (Zhang YJ et al. 2016; Zhang Y et al. 2020). Chloroplast genome has been evidenced to be effective in plant phylogeny and species identification (Yang et al. 2013). In this study, we sequenced the complete chloroplast genomes of *Epimedium muhuangense*, aiming to provide valuable information for taxonomic and phylogenetic studies on the genus *Epimedium*.

In this study, *E. muhuangense* samples were collected from its type locality, Muhuang Town, Yinjiang County, Guizhou, China (E108°40′, N28°5′). A specimen was deposited at the Herbarium of the Institute of Wuhan Botanical Garden, Chinese Academy of Medical Science (http://www.whiob.ac.cn/, Yanjun Zhang, yanjunzhang@wbgcas.cn) under the voucher number Yanjun Zhang 568. The genomic DNA was extracted from fresh leaves using the modified CTAB method (Doyle and Doyle 1987). The chloroplast genome was sequenced using Illumina Novaseq PE150. The sequenced clean reads were further assembled using the program GetOrganelle v1.7.4.1 (Jin et al. 2020) with *E. acuminatum* chloroplast genome (GenBank accession number: NC_029941) as a reference. The gene annotation was performed by online programs Geseq (Michael et al. 2017) and CPGAVAS2 (Shi et al. 2019), followed by manual correction. The chloroplast genome sequence of *E. muhuangense* was submitted to NCBI database with an accession number (OK166811).

The chloroplast genome of *E. muhuangense* was 157,264 bp in length, consisting of a small single copy region (SSC, 17,036 bp), a large single copy region (LSC, 88,588 bp), and two inverted repeat regions (IRa and IRb, 25,820 bp). The total GC content of chloroplast genomes was 38.77%, while the content of GC in LSC, IR, and SSC regions was 37.37%, 43.17%, and 32.77%, respectively. The chloroplast genome of *E. muhuangense* contains 112 unique genes, including 78 protein-coding genes, 30 tRNA genes, and four rRNA genes. Among these genes, 15 genes (six tRNA genes and nine protein-coding genes) contained an intron, and three genes contained a pair of introns. In addition, five protein-coding genes (ndhB, rpl23, rps7, rps12, ycf2), eight tRNA genes (trnI-CAU, trnI-GAU, trnL-CAA, trnN-GUU, trnA-UGC, trnR-ACG, trnV-GAC, and trnQ-UUG) and four rRNA genes (rrn4.5, rrn5, rrn16, and rrn23) were duplicated to have double copies in the chloroplast genome.

To explore the phylogenetic position of *E. muhuangense*, the complete chloroplast genome sequences of 15 plant species were downloaded from the NCBI GenBank database. Sequences were aligned using MAFFT v.7 (Katoh et al. 2019) and trimmed using MEGA v.11 (Tamura et al. 2021). A maximum likelihood tree was constructed using raxmlGUI 2.0 (Edler et al. 2021) with *Vancouveria hexandra* (Hook.) C. Morren & Decne as outgroup (Figure 1). The results of the phylogenetic analysis show that *E. muhuangense* is closely related to *E. elachyphyllum*, both of which are distributed in northeastern Guizhou. Furthermore, *E. muhuangense* and *E. elachyphyllum* are the only two species only having unifoliolate leaves in *Epimedium* (Zhang YJ et al. 2011; Wang et al. 2017). The chloroplast genome of *E. muhuangense* will contribute to the research of phylogeny and evolution of Berberidaceae.

**Figure 1.**
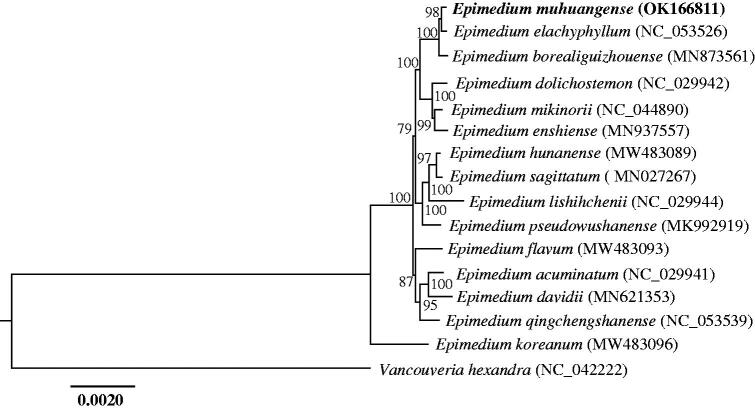
The maximum likelihood (ML) phylogenetic tree based on complete chloroplast genomes of 16 species, with *Vancouveria hexandra* as outgroup. Numbers at nodes represent bootstrap values.

## Authors’ contributions

Jing Wang and Ruoqi Huang designed and performed the experiments; Jing Wang also analyzed the data and drafted the manuscript; Qiong Liang and Yanjun Zhang revised critically for intellectual content and approved the final version of the paper; and all authors agree to be accountable for all aspects of the work.

## Ethical approval

The plant materials used in this study were transplanted into Wuhan Botanical Garden for cultivation through legal collection way. And the study was approved by the Wuhan Botanical Garden of the Chinese Academy of Sciences.

## Data Availability

The genome sequence data that support the findings of this study are openly available in GenBank of NCBI at (https://www.ncbi.nlm.nih.gov/) under the accession no. OK166811. The associated BioProject, SRA, and Bio-Sample numbers are PRJNA774127, SRR16571520, and SAMN22555212, respectively.

## References

[CIT0001] Doyle JJ, Doyle JL. 1987. A rapid DNA isolation procedure for small quantities of fresh leaf tissue. Phytochem Bull. 19(1):870–15.

[CIT0002] Edler D, Klein J, Antonelli A, Silvestro D. 2021. raxmlGUI 2.0: a graphical interface and toolkit for phylogenetic analyses using RAxML. Methods Ecol Evol. 12(2):373–377.

[CIT0003] Guo MY, Pang XH, Xu YQ, Jiang WJ, Liao BS, Yu JS, Xu J, Song JY, Chen S. 2022. Plastid genome data provide new insights into the phylogeny and evolution of the genus *Epimedium*. J Adv Res. 36:175–185. 35127172 10.1016/j.jare.2021.06.020 PMC8799909

[CIT0004] Jin JJ, Yu WB, Yang JB, Song Y, dePamphilis CW, Yi TS, Li DZ. 2020. GetOrganelle: a fast and versatile toolkit for accurate de novo assembly of organelle genomes. Genome Biol. 21(1):241. 32912315 10.1186/s13059-020-02154-5 PMC7488116

[CIT0005] Katoh K, Rozewicki J, Yamada KD. 2019. MAFFT online service: multiple sequence alignment, interactive sequence choice and visualization. Brief Bioinform. 20(4):1160–1166. 28968734 10.1093/bib/bbx108 PMC6781576

[CIT0006] Luo H, Liang Q, Huang RQ, Dong JZ, Zhang YJ. 2021. The complete chloroplast genome of *Epimedium flavum* Stearn (Berberidaceae). Mitochondrial DNA B Resour. 6(9):2484–2485. 34377801 10.1080/23802359.2021.1920500 PMC8330771

[CIT0007] Michael T, Pascal L, Tommaso P, Ulbricht-Jones ES, Axel F, Ralph B, Stephan G. 2017. GeSeq – versatile and accurate annotation of organelle genomes. Nucleic Acids Res. 45(W1):W6–W11. 28486635 10.1093/nar/gkx391 PMC5570176

[CIT0008] Shi L, Chen H, Jiang M, Wang L, Wu X, Huang L, Liu C. 2019. CPGAVAS2, an integrated plastome sequence annotator and analyzer. Nucleic Acids Res. 47(W1):W65–W73. 31066451 10.1093/nar/gkz345 PMC6602467

[CIT0009] Tamura K, Stecher G, Kumar S. 2021. MEGA11 molecular evolutionary genetics analysis version 11. Mol Biol Evol. 38(7):3022–3027. 33892491 10.1093/molbev/msab120 PMC8233496

[CIT0010] Wang YY, Xu WF, He SZ. 2017. *Epimedium muhuangense* (Berberidaceae), a new species from China. Phytotaxa. 319(3):277–282.

[CIT0011] Yang JB, Tang M, Li HT, Zhang ZR, Li DZ. 2013. Complete chloroplast genome of the genus *Cymbidium*: lights into the species identification, phylogenetic implications and population genetic analyses. BMC Evol Biol. 13:84. 23597078 10.1186/1471-2148-13-84 PMC3644226

[CIT0012] Zhang YJ, Dang HS, Wang Y, Li XW, Li JQ. 2011. A taxonomic revision of unifoliolate Chinese *Epimedium* L. (Berberidaceae). Kew Bull. 66(2):253–262.

[CIT0013] Zhang YJ, Du LW, Liu A, Chen JJ, Wu L, Hu WM, Zhang W, Kim K, Lee SC, Yang TJ, et al. 2016. The complete chloroplast genome sequences of five *Epimedium* species: lights into phylogenetic and taxonomic analyses. Front Plant Sci. 7:12. 27014326 10.3389/fpls.2016.00306 PMC4791396

[CIT0014] Zhang Y, Liu X, Yao Y, Luo Y, Yang Q, Zhang C, Chaoqun X, Suo F, Shen G, Guo B. 2020. The complete chloroplast genome of *Epimedium pudingense* (Berberidaceae), a narrowly distributed plant species in China. Mitochondrial DNA B Resour. 5(3):2631–2633. 33457886 10.1080/23802359.2020.1781560 PMC7782884

[CIT0015] Zhou M, Zheng W, Sun XG, Yuan M, Zhang J, Chen XJ, Yu KT, Guo BL, Ma BP. 2021. Comparative analysis of chemical components in different parts of *Epimedium* Herb. J Pharm Biomed Anal. 198:113984. 33691203 10.1016/j.jpba.2021.113984

